# A land-use benefit evaluation system with case study verification

**DOI:** 10.1371/journal.pone.0271557

**Published:** 2022-07-29

**Authors:** Haiyuan Sun, Linlin Cheng, Zhuo Li, Qiyuan Wang, Jiahua Teng

**Affiliations:** 1 College of Geoscience and Surveying Engineering, China University of Mining and Technology (Beijing), Beijing, China; 2 College of Land Science and Technology, China Agricultural University, Beijing, China; 3 Ministry of Ecology and Environment Center for Satellite Application on Ecology and Environment, Beijing, China; Gebze Teknik Universitesi, TURKEY

## Abstract

In regional land-use planning, many different demands for often-limited land resources must be weighed against each other. Analysis of the benefits of different land-use types is of great significance in land-use design. However, a good evaluation methodology does not exist. To facilitate a comparative analysis of land-use benefits, this paper presents an evaluation system consisting of four steps: (1) Connotation dissection to determine the land-use benefits, (2) construction of a land-use benefit classification system to summarize a limited number of land-use benefit types by an inductive method, (3) land-use benefit valuation, which includes a biophysical model, direct and indirect market valuations, and The Economics of Ecosystems and Biodiversity value conversion method, and (4) case analysis of the evaluation results according to local conditions. Empirical results from a case study of Mentougou District, Beijing, China, show that (i) the evaluation results of land-use benefit groups provides information on each land-use type and the spatial distribution of land-use benefits in Mentougou District, (ii) the topography of Mentougou District has an important influence on economic and ecological land-use benefits, and (iii) there is a synergistic effect of economic and social land-use benefits.

## 1 Introduction

Land is a basic requirement for human existence and has great ecological, socio-cultural and economic value [1]. In regional land-use planning and decision-making, specific land-use arrangements and layouts are often made to achieve specific objectives [2, 3]. Many different demands for often-limited land resources must be weighed against each other. In such decision processes, comprehensive and objective evaluations of the economic, social, and ecological benefits of land use can provide a scientific foundation for land-use design.

There has been a number of fruitful studies in this field. The methods employed include not only conventional methods such as analytic hierarchy process (AHP), expert consultation, artificial neural net (ANN), and fuzzy comprehensive evaluation method (FCE), but also new methods such as geographic information system(GIS), remote sensing technology and spatial econometric models [4–9].The application of the new methods pays more attention to visual analysis [10]. The perspective of land-use benefit evaluation often focusses on a single or selected several aspects of land use [11, 12]. Andrea analyzed flood-prone land-use benefits by the income per unit area from seasonal agriculture and the net income per fisherman from wild fish capture in Candaba, Philippines [13]. Luan evaluated the spatial and environmental benefits of green space ecosystem services in a local rural context [14]. These depend upon the observational perspective used, which in turn depends on the observer’s analytical purpose.

Scholars’ studies have laid a solid foundation for follow-up research, but the land-use benefit evaluation system for land-use planning has not emerged. There are several reasons for this phenomenon [15, 16]. First, and perhaps most importantly, land-use planning is multi-objective and difficult to evaluate from multiple perspectives. This is due to the diversity of regional socio-economic development needs. Second, ecological and social benefits have public attributes, which are not captured in conventional, market-based economic analyses. A final reason is the multi-disciplinary nature of land. It is difficult for a certain discipline to deeply study all of the benefits, and we need to overcome many obstacles and cross many disciplinary bridges. Based on the above analysis, this paper dissects the meaning of land-use benefits, in which perspectives of related disciplines needed to construct a land-use benefit classification system are identified. Then, a land-use benefit evaluation system is reconstructed by an inductive method and applied to Mentougou District of Beijing.

## 2 The basic connotation of land-use benefits

**Fig 1:**
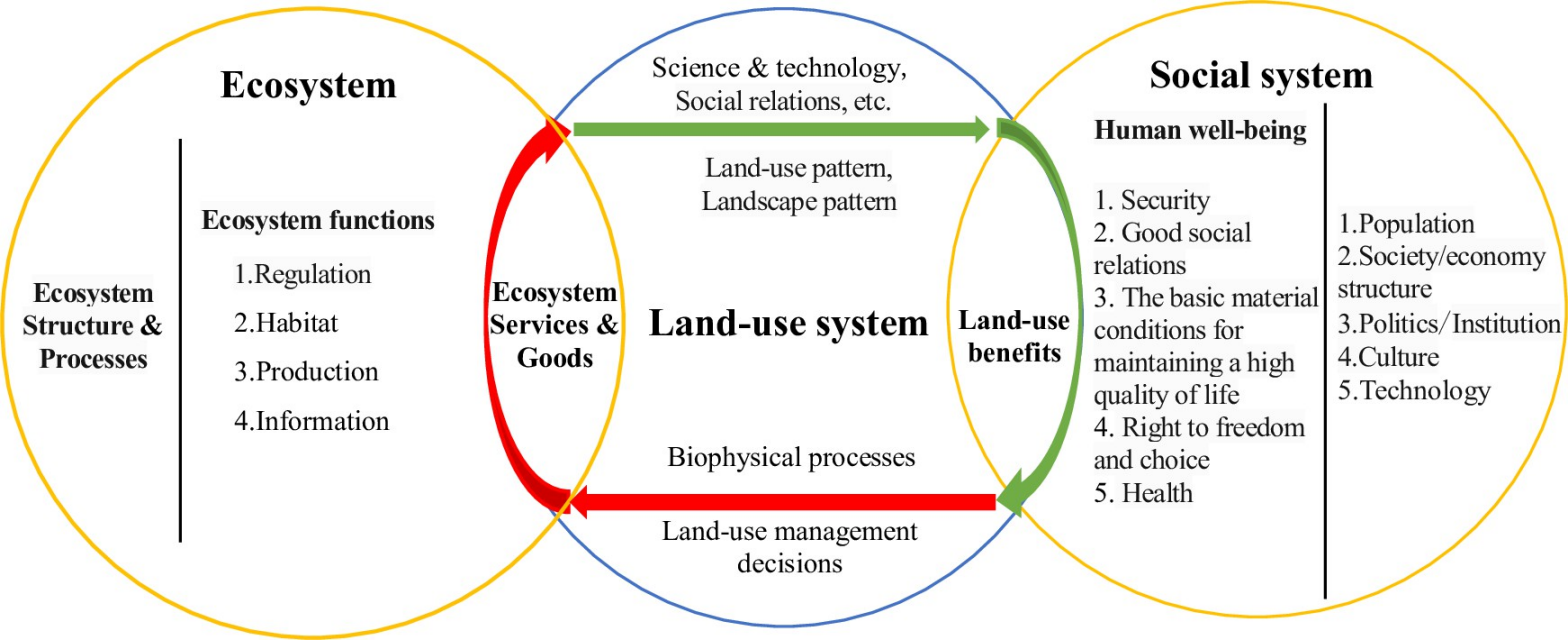
Analysis framework of the basic connotations of land-use benefits.

From the beginning of agrarian society and through the industrial society, to the post-industrial society, humankind has changed land-use to improve the amount, quality, and security of natural resources. The sustainable development of human society not only relies on the supply capacity of land but also on the coordination of land-use functions [17, 18]. As a complex nature-society-economy system, land carries out the matter cycle, information transfer, and energy flow within itself and with the surroundings. It is on the basis of these artificial or natural ecological processes that the various services that land is endowed with are derived. *Land-use benefits*, therefore, can be defined as the goods and services obtained from land-use. This description is further interpreted in Fig 1.

As shown in Fig 1, ecosystems consist of microbes, plants, animals, and the abiotic environment. They have multilayer structures and complex processes that are often classified into a limited number of ecosystem functions: i.e., regulation, habitat, and production [1, 15, 16]. These ecosystem processes provide, directly or indirectly, the services (e.g., climate regulation, aesthetic information, waste treatment) and goods (such as food, timber, minerals) necessary for the sustainable development of human society. Land-use is an important way for human beings to attain services and goods from the ecosystem, which are known as *land functions* and *landscape functions* [1, 15, 19]. In the light of the purposes and natural characteristics of land, human beings take biological and physical measures to carry out long-term and periodic management of land to actively or passively obtain ecosystem services and goods. For example, in agro-ecosystems, humans actively obtain agro- products for food through land cultivation techniques, and agro-ecosystems also provide humans with water regulation, waste treatment, and so on. Human being, directly or indirectly, transforms these services and goods into human well-being- i.e., security, good social relations, the basic material conditions for maintaining a high quality of life [17, 20]. Yet, mankind also promotes the evolution of the ecosystems to better serve human beings through land-use decision-making and coupling of biophysical processes, ensuring that ecosystems sustainably contribute to human well-being.

## 3 Land-use benefit evaluation system

### 3.1 Construction framework of the land-use benefit classification system

**Fig 2:**
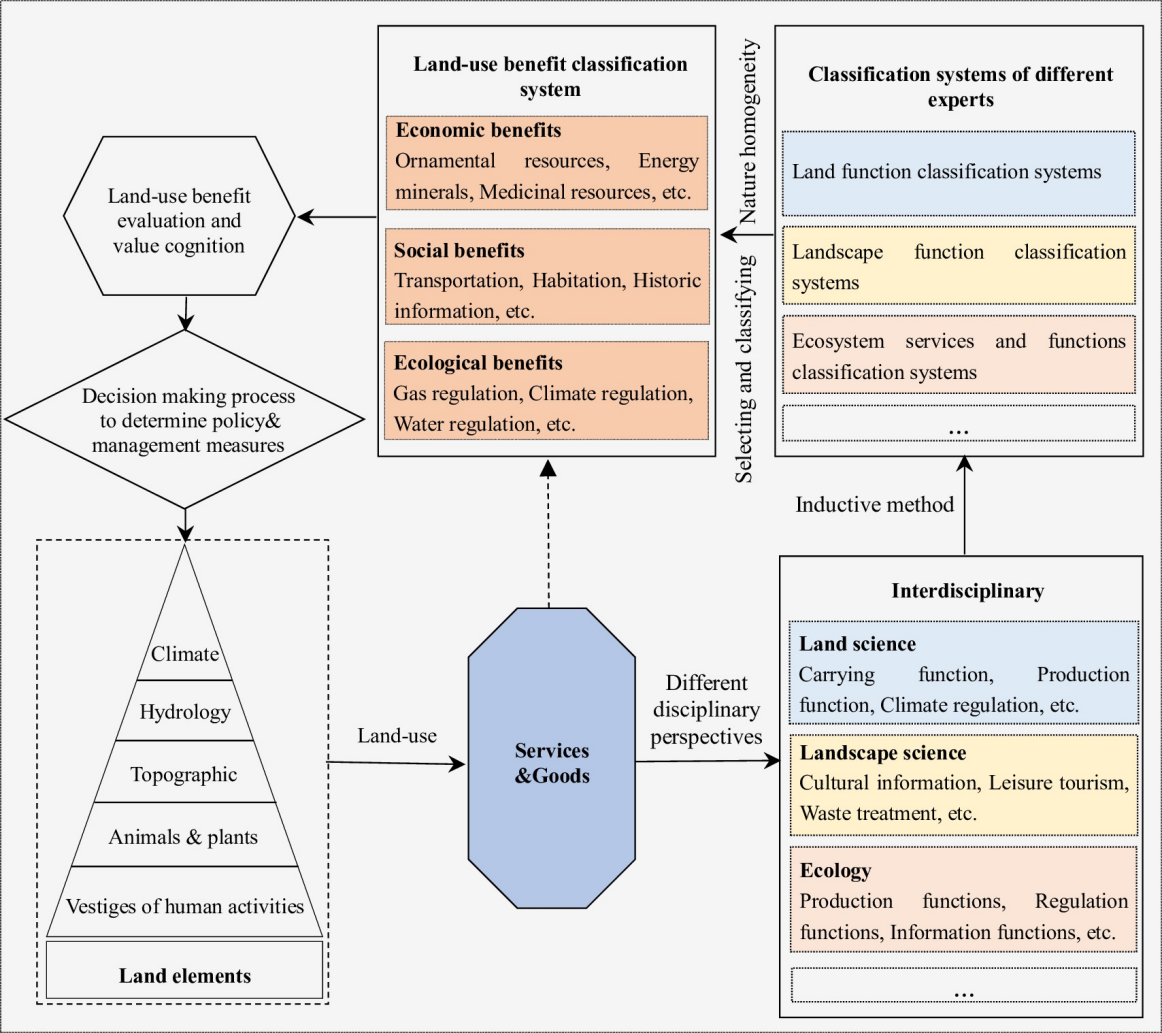
Construction framework of the land-use benefit classification system.

Land-use is an important way for human beings to attain services and goods in order to meet human needs (Fig 2). From the perspective of land science, ecology, and landscape science, land-use benefits are indirectly or directly reflected in the ecological, economic, and social functions of land, landscape functions, and the ecosystem goods and services provided by the ecosystem [1, 15, 16]. Therefore, an inductive method was employed in our study to summarize the existing classifications of ecosystem goods and services land functions, and landscape functions. Then, land-use benefit types were selected from the summarized results in line with the types of human well-being, and were merged according to the principle of natural homogeneity. Finally, the land-use benefit types were divided into economic, social, and ecological benefits according to the differences in their characteristics.

#### (1) The interdisciplinary basis of the construction of land-use benefit classification system

Ecosystem services has attracted the attentions of many scholars and research projects since the concept was introduction in the 1970’s [1, 16, 18, 21–23]. According to Costanza, ecosystems have 17 functions [16]. De Groot divided ecosystem functions into four main categories: regulation, habitat, production, and information, which are further divided into 23 specific functional sub-categories [21]. Millennium Ecosystem Assessment (MA) also divides ecosystem functions into four main categories (regulation, habitat, production, and information functions) and 28 functional sub-categories, which are similar to de Groot’s categories [23]. While there are many differences in ecosystem function classifications, all valuations of ecosystem services are based on land-use types and their areas. Therefore, land-use types, patterns, and intensity are widely recognized as the important factors to estimating the value of ecosystem services.

A landscape is a spatially heterogeneous mosaic of natural, societal, and economic elements. A landscape pattern formed by the arrangement and combination of different land-use types generates corresponding ecological processes that affect ecosystem’s material migration, energy flows, and information transfer. Landscape functions, essentially, are vitally related to ecosystem functions, and can be used to describe different kinds of landscape goods and services. Based on research on ecosystem services, de Groot divided landscape functions into regulation, habitat, production, information, and carrier functions, and further divided them into 23 specific functional sub-categories [15]. Lovell divided landscape functions into three main categories from the perspective of landscape multifunctionality [24]. Willemen classified the landscape functions into residential, intensive livestock, drinking water, cultural heritage, tourism, plant habitat, arable production, and leisure cycling from the view of multiobjective programming [19].

*Land function* refers to the capacity of land to provide goods and services [25]. Yet, no comprehensive and unified land function classification system has emerged. Paracchini attempted to classify land functions into three broad basic categories—economic, environmental, and social functions- and nine specific subcategories [26]. Based on the characteristics of human activities, Chinese scholars and government agencies classify land functions into three main types: production, living and ecological functions [27]. From the perspective of economics, land functions can be classified into seven categories [28]. Zhang classified land functions into ten subcategories on the basis of production, living, ecological functions [29].

#### (2) Land-use benefit classification system

*Ecological benefits* refer to the beneficial effects of services and goods on the environment. Consequently, gas regulation, climate regulation, water regulation, nutrient regulation, pollination, species diversity, net primary productivity, soil retention, disturbance prevention, and waste treatment were classified as land-use ecological benefits. *Economic benefits* are the direct economic values of products and services in transactions. As a result, water, food, medicinal resources, raw materials, ornamental resources, energy minerals, and products and services were classified as land-use economic benefits. *Social benefits* that have no explicit markets refer to the beneficial effects of services on the quality of human experience. Habitation, transportation, employment security, basic living security, recreation and aesthetic information, historic information, science, education, cultural and artistic information were classified as land-use social benefits. See Table 1 for details.

**Table 1:** Land-use benefit classification system.

Land-use benefit types		Explanations	Characterizations	Equations	References
Land-use economic benefits	Water	Retention and storage of water	The amount of freshwater available to people now or in the future	(1-1)–(1-5)	[15, 16, 21]
	Food	Food such as cereals and potatoes, fungus, poultry meat product, etc., obtained from cultivated crop or captive animal, obtained by hunting animals, picking plants and fungus, and fishing, etc.	The supply of food products, livestock, fishery products, wildlife products, etc.	(2-1)	[18, 19]
	Medicinal resources	Variety in (bio)chemical sub-stances in, and other medicinal uses of, natural biota	The supply of natural drugs, derived drugs, and synthetic drugs	(3-1)	[15, 21, 23]
	Raw materials	Biological resources such as timber and fiber, energy resources, forage, biodynamic compounds, etc.	The supply of fiber, timber, forage, hides, and skins, etc.	(4-1)	[16, 21, 23]
	Ornamental resources	Ornamental flora, fauna, woodwork, shells, etc.	Natural materials for craftwork, ornamental horticultural plants and fish etc.	(3-1)	[15, 18, 21]
	Energy minerals	Petroleum, natural gas, natural minerals or ore, etc., buried in the ground or distributed on the surface.	The production of petroleum, natural gas, coal, rare earth, copper minerals, metallic mineral, etc.	(5-1)	[15, 28]
	Products and services	Final products and services provided by secondary or tertiary industries	The gross output value of commerce and services, and industry	(6-1)	[27, 28]
Land-use social benefits	Habitation	Carrying housing and its ancillary facilities	The base price of residential land	(7-1)–(7-2)	[15, 19, 26]
	Transportation	Carrying transport routes and facilities	The gross output value of transportation	(8-1)	[15, 29]
	Recreation and aesthetic information	Providing space for leisure travel	The largest number of tourists carried	(9-1)–(9-2)	[21, 23]
	Historic information	Recording the information of nature, religion, history, culture, etc.	The number of historical prints.	(9-1)–(9-2)	[20, 24, 26]
	Science, education, cultural and artistic information	Offering targets and places for scientific research and education; providing objects and inspiration for art	The number of scientific research projects, the number of educational courses, the number of aesthetic prints, etc.	(9-1)–(9-2)	[16, 21]
	Employment Security	Guarantee of employment	The wages of guaranteed employees	(10-1)–(10-2)	[26, 28]
	Basic living security	Provision of daily necessities	Standard of minimum living standard for urban residents	(10-1)–(10-2)	[28]
Land-use ecological benefits	Gas regulation	Maintaining biological and geochemical processes of the atmosphere	The ability to regulate the atmospheric composition	(11-1)	[16, 18, 21]
	Climate regulation	Regulating regional and global climate processes	The ability to regulate climate	(12-1)–(12-3)	[16, 18, 21]
	Water regulation	Role of land cover in regulating runoff & river discharge	The amount of water conservation	(13-1)–(13-2)	[16, 18, 21]
	Nutrient regulation	Regulating regional and global nutrient (N, S, and P, etc.) cycle	The value of nutrient cycle	(14-1)	[16, 18, 21]
	Pollination	Role of biota in the movement of floral gametes	The species and distribution of plants, availability of pollinators	(15-1)–(15–2)	[16, 18, 21]
	Species diversity	Maintaining the existence and succession of the gene pool	Species diversity and sustainability	(4-1)	[16, 18, 21]
	Net primary productivity	The primary production capacity of the plant	Net primary production capacity	(16-1)–(16–9)	[18]
	Soil retention	Retarded soil erosion and landslides through vegetation roots, etc.	The amount of soil conservation	(17-1)–(17-11)	[16, 21]
	Disturbance prevention	Suppression and amelioration of sudden event (floods, fires, hurricanes, earthquakes, etc.) disturbances	The cost of storm protection, flood prevention, disease control, etc.	(18-1)	[15, 18, 23]
	Waste treatment	Purifying the pollutants, toxins, etc. of water and air by the way of dilution, assimilation, and recombination	Waste purification values of air and water	(19-1)–(19–3)	[15, 18]

### 3.2 Methodological system of land-use benefit evaluation

There are established methods for evaluating 19 of the types of land-use benefits, but none for the following five: historic information, recreation and aesthetic information, medicinal resources, ornamental resources, science, education, cultural and artistic information. Therefore, we used the The Economics of Ecosystems and Biodiversity (TEEB)—value conversion method to evaluate them.

#### (1) Water

The amount of water refers to the amount of water that runs off the landscape. A combination of a modeling technique and outcome parameter method is employed to evaluate the benefits of water supply capacity per land use type. The equations are as follows [30, 31]:

$$V_{x}=(1-{AET}_{x}/P_{x})∙TW∙P_{x}$$ (1-1)

AETx/Px=(1+wxRx)/[1+wxRx+(1Rx)] (1-2)

$$w_{x}=({AWC}_{x}/P_{x})∙Z$$ (1-3)

$$R_{x}=k_{x}{ETO}_{x}/P_{x}$$ (1-4)

where *V*_*x*_ is the value of water supply provided by land-use type *x* per area annually, *AET*_*x*_ is the annual actual evapotranspiration, *TW* is the price of fresh water, *P*_*x*_ is average annual precipitation, *w*_*x*_ is the corrected value of vegetation cover, *R*_*x*_ is Budyko dryness index, *AWC*_*x*_ is the available soil moisture, Z is a seasonal rainfall factor, *k*_*x*_ is the evapotranspiration coefficient, *ETO*_*x*_ is the reference evapotranspiration. For the relevant parameter settings, refers to Leh [30].

#### (2) Food

Food supplying capacity is defined as the amount of food that agricultural land can grow annually. A realistic productivity potential model is employed in this paper to evaluate the benefits of food supplying capacity, and its equation as follows:

$$V_{x}=\sum Y_{xj}∙\lambda (M){∙T}_{j}$$ (2-1)

where *V*_*x*_ is the crop value provided by land-use type *x* per area annually, *Y*_*xj*_ is the crop *j* productivity of land-use type *x*, λ(M) is the social effectiveness coefficient, *T*_*j*_ is the price of crop *j*.

#### (3) Medicinal resources and ornamental resources

There are now no established methods for measuring these two types of land-use benefits. Therefore, this paper uses the TEEB-value conversion method to evaluate them, and the equation as follows:

$$V_{x}=V_{mean(x)}∙S_{x}/S$$ (3-1)

where *V*_*x*_ is the medicinal resources value or ornamental resources value provided by land-use type *x* per area annually. *V*_mean(*x*)_ is the global statistical value of medicinal resources or ornamental resources of TEEB provided by land-use type *x* per area annually, *S*_*x*_ is the area of land-use type *x* used to plant medicinal material or produce ornamental resources, and *S* is the area of land-use type *x*.

#### (4) Raw materials and species diversity

Raw materials and species diversity are difficult to evaluate because of its varying definitions and perceptions. The dynamic evaluation method for ecosystem service value proposed by Xie is employed in this paper to evaluate the benefits of raw materials supply capacity and maintenance of species diversity. The equation is as follows [22]:

$$V_{x}=F_{x}∙(NPP/\bar{NPP})∙(T/\bar{T})∙CE$$ (4-1)

where *V*_*x*_ is the raw materials value or value of maintaining biodiversity provided by land-use type *x* per area annually, *F*_*x*_ is the equivalent factor of land-use type *x*, *NPP* is the average Net primary productivity for study site’s land-use type *x* in the target year, $\bar{NPP}$ is the average NPP of Chinese ecosystems in 2010, *T* is the mean price of study site’s grain in the target year, $\bar{T}$ is the mean price of Chinese grain in 2010, *CE* represents the value of ecosystem services in 2010 for one standard equivalence factor (set to 3406.5 yuan/hm^2^). For the relevant parameter settings, refer to Xie [22].

#### (5) Energy minerals

Due to the availability of data on energy minerals, direct market valuation can be employed to evaluate them. The equation is as follows:

$$V_{x}=\sum (Y_{i}∙T_{i})/S_{x}$$ (5-1)

Where *V*_*x*_ is the energy minerals value provided by land-use type *x* per area annually, *Y*_*i*_ is the case area’s yield of minerals or energy sources *i*, *T*_*i*_ is the price of minerals or energy sources *i*, *S*_*x*_ *is* the area of land-use type *x* used for minerals or energy sources production.

#### (6) Products and services

Products and services are often considered the final products and services of secondary or tertiary industries. There are explicit markets for products and services, and direct market valuation can be used to calculate the value of products and services as:

$$V_{x}=\sum G_{x}/S_{x}$$ (6-1)

where *V*_*x*_ is the value of products and services provided by land-use type *x* per area annually, *G*_*x*_ is the gross domestic product of secondary industry and tertiary industries provided by land-use type *x*, and *S*_*x*_ *is* the area of land-use type *x*.

#### (7) Habitation

Habitation means that the land carries residential facilities. This paper uses a land capitalization approach to evaluate it with the following equations:

$$V_{x}=T_{x}∙\left[\frac{Sum∙t}{S_{x}}\right]∙{MD}_{x}$$ (7-1)

$${MD}_{x}=\left\{\sum_{r=0}^{N_{x}-1}\left\{\left[{1-1/(1+\alpha_{x})}^{N_{x}-r}]/[{1-1/(1+\alpha_{x})}^{N_{x}}\right]\right\}\right\}/N_{x}$$ (7-2)

where *V*_*x*_ is the habitation value of land-use type *x* per area annually, *T*_*x*_ is the base price of urban land or the compensation standard for land expropriation of land-use type *x*, *Sum* is the regional urban population or rural population, *t* is the area of urban land or rural residential land per capita, *S*_*x*_ is the area of urban land or rural residential land, *MD*_*x*_ is the mean depreciation ratio of land-use type *x*, *N*_*x*_ is the design life of a house, *r* is a integral number(0≤*r*≤*N*_*x*_−1), and *α*_*x*_ is the depreciation ratio of land-use type *x*.

#### (8) Transportation

Transportation indicates that land can provide a suitable substrate for transportation facilities. Direct market valuation is adopt to evaluate it, and it is computed as:

$$V_{x}=GP/S_{x}$$ (8-1)

where *V*_*x*_ is the transportation value provided by land-use type *x* per area annually, *GP* is the regional gross product of transportation, *S*_*x*_ is the area of transportation land.

#### (9) Recreation and aesthetic information, historic information, science, education, cultural and artistic information

These reveal opportunities for cognitive development. TEEB-value conversion method and travel cost are used to evaluate it [21]. The equations are:

$$V_{x1}=O/S$$ (9-1)

$$V_{x2}=V_{mean}∙\beta$$ (9-2)

where *V*_*x*1_ is the value of recreation and aesthetic information provided by land-use type *x*1 per area, *O* is the operating revenue of scenic institutions, *S* is the area of scenic sites, *V*_*x*2_ is the value of historic information or science, education, cultural and artistic information provided by land-use type *x*2 per area annually, *V*_mean_ is the global statistical value of TEEB of historic information or science, education, cultural and artistic information, and *β* is the region correction coefficient.

#### (10) Employment security and basic living security

Due to the attributes of public land used for employment security and basic living security services, there are no explicit markets for their value. This paper uses replacement cost to evaluate them. The equations are as follows:

$$V_{x1}=({sum}_{x1}∙H_{x1})/S_{x1}$$ (10-1)

and

$$V_{x2}={sum}_{x2}∙I$$ (10-2)

where *V*_*x*1_ is the employment security value provided by land-use type *x*1 per area annually, *sum*_*x*1_ is the employment population of industry carried by land-use type *x*1, *H*_*x*1_ is the average wage of industry carried by land-use type *x*1, *S*_*x*1_ is the area of land-use type *x*1, *V*_*x*2_ is the basic living security value provided by land-use type *x*2 per area annually, *sum*_*x*2_ is the population supporting capacity of cultivated land, and *I* is the minimum living standard for urban residents.

#### (11) Gas regulation

Vegetation contributes to the carbon cycle by photosynthesis, which maintains the dynamic balance of CO_2_ and O_2_ in the atmosphere. In this paper, we derive a gas regulation valuation model based on the photosynthesis equation, and then combine it with the carbon tax and industrial oxygen-producing method to assess the value of gas regulation services. The equation is as follows [32]:

$$V_{x}=1.62∙{NPP}_{x}∙T_{1}+1.2∙{NPP}_{x}∙T_{2}$$ (11-1)

where *V*_*x*_ is the gas regulation value provided by land-use type *x* per area annually, *NPP*_*x*_ is the annual production of net primary productivity per area of land-use type *x*, *T*_1_ is carbon tax rate, and *T*_2_ is the cost of industrially manufactured oxygen.

#### (12) Climate regulation

Land cover and biology maintain a favorable climate for human. Because there are no explicit markets for this service, replacement cost can be a indirect means of assessing the climate regulation values. The equations are as follows [33]:

$$V_{x}=V_{x1}+V_{x2}$$ (12-1)

$$V_{x1}=J_{x1}∙\alpha ∙\rho ∙T_{e}$$ (12-2)

$$V_{x2}=J_{x2}∙\gamma ∙\rho ∙T_{e}$$ (12-3)

where *V*_*x*_ is the climate regulation value provided by land-use type *x* per area annually, *V*_*x*1_ is the value of transpiration, *V*_*x*2_ is the value of evaporation, *J*_*x*1_ is the amount of heat absorbed by green space per unit, *α* is the effectiveness ratio of air conditioning, *ρ* is a constant (1kWh/3600kJ), *T*_*e*_ is the electricity price, *J*_*x*2_ is the evaporation capacity of the water surface, and *γ* is the amount of heat consumed to evaporate a unit volume of water.

#### (13) Water regulation

Water regulation service is mainly defined as the regulation of hydrological flows at the earth surface by land-cover, maintaining ‘normal’ conditions [34, 35]. As there is no exchange value in trade, avoided cost can be used in this paper to evaluate it. The equations are as follows [35, 36]:

$$V_{x}=Q_{x}∙CR$$ (13-1)

$$Q_{x}=\epsilon ∙P_{x}+G_{x}∙L_{x}+D_{x}∙A+I_{x}$$ (13-2)

Where *V*_*x*_ is the water regulation value provided by land-use type *x* per area annually, *Q*_*x*_ is the amount of conserve water in land-use type *x* per area, *CR* is the construction cost of the unit reservoir storage, *ϵ* is the rate of rainfall intercepted by vegetation, *P*_*x*_ is average annual precipitation, *G*_*x*_ is the dry weight of forest floor, *L*_*x*_ is field water-retaining capacity of the forest floor, *D*_*x*_ is the vertical extent of soil, *A* is the soil porosity, and *I*_*x*_ is the surface water storage. For the relevant parameters setting, refer to Mo [35].

#### (14) Nutrient regulation

Nutrient regulation service indicates the amount of nutrients that can be used by humans in nutrient cycling. It is represented by the nitrogen, phosphorus, potassium and organic matter content of NPP. A replacement cost is used to evaluate it. The equation is as follow [37]:

$$V_{x}={NPP}_{x}∙(NC∙T_{3}/R_{1}+PC∙T_{4}/R_{2}+KC∙T_{4}/R_{3}+MC∙T_{5})$$ (14-1)

where *V*_*x*_ is the nutrient regulation value provided by land-use type *x* per area annually, *NPP*_*x*_ is the annual production of net primary productivity per area in land-use type *x*, *NC*, *PC*, *KC*, and *MC* are the nitrogen, phosphorus, potassium and organic matter content of the *NPP*_*x*_ respectively, *T*_3_, *T*_4_, *T*_5_ are the prices of ammonium phosphate, potash fertilizer, and organic fertilizer respectively, *R*_1_, *R*_2_, *and R*_3_ are the rates of *NC* used for ammonium phosphate, *PC* used for ammonium phosphate, and *KC* used for potash fertilizer, respectively.

#### (15) Pollination

Pollination is indispensable for most plants to breed. Currently, quantitative research on the pollination is still in its infancy and is impeded by the complexity of this ecological process. A combination of a modeling method proposed by Robinson and outcome parameter method model is used to assess the value of pollination. The equations are as follows [38, 39]:

$$V_{x}=\sum_{\omega =1}^{\tau }Y_{\omega x}∙D_{\omega }∙T_{\omega }$$ (15-1)

$$D_{\omega }=(Y_{\omega 0}-Y_{\omega c})/Y_{\omega o}$$ (15-2)

where *V*_*x*_ is the pollination value provided by land-use type *x* per area annually, *τ* is the number of crop species grown in the land-use type *x*, *ω* stands for the *i*th crop (1≤*ω*≤*τ*), *Y*_*ωx*_ is the crop’s production of land-use type *x* per area, *D*_*ω*_ is the degree of crop’s dependency on insects, *T*_*ω*_ indicates agricultural price, *Y*_*ω*0_ is the pollinated crop’s production in open cropland, and *Y*_*ωc*_ is the crop’s production in cropland without insects. For relevant parameters setting, refer to Liu [39].

#### (16) Net primary productivity (NPP)

Almost all animals and micro-organisms on Earth rely directly or indirectly on net primary production from the photosynthesis of plants. The CASA model is based on remote sensing data and is currently one of the most commonly used models for estimating NPP, its equations are as follows [40–42]:

$$V_{x}={NPP}_{x}∙T_{c}∙1.2$$ (16-1)

$${NPP}_{x=}{FAPAR}_{x}∙\varepsilon_{\max\left(x\right)}∙\varepsilon_{x1}∙\varepsilon_{x2}∙\varepsilon_{x}$$ (16-2)

where *V*_*x*_ is the NPP value provided by land-use type *x* per area annually, *NPP*_*x*_ is the net primary productivity, *T*_*c*_ is the price of standard coal, *FAPAR*_*x*_ is the photosynthetically active radiation, *ε*_max(*x*)_ is the maximum efficiency of solar energy utilization, *ε*_*x*1_ and *ε*_*x*2_ are the coefficients of low- and high-temperature stress, respectively, and *ε*_*x*_ is the coefficient of water stress. *FAPAR*_*x*_, *ε*_max(*x*)_, *ε*_*x*1_, *ε*_*x*2_, and *ε*_*x*_ are given as:

$${FAPAR}_{x}=0.00949∙0.5∙{SOL}_{x}∙\left[\frac{{SR}_{x}-{SR}_{\min\left(x\right)}}{{SR}_{\max\left(x\right)}-{SR}_{\min\left(x\right)}}\right]$$ (16-3)

$$\varepsilon_{x1}=0.8+0.02∙{opt}_{x}-0.0005∙{{opt}_{x}}^{2}$$ (16-4)

$$\varepsilon_{x2}=\left\{\frac{1.184}{\left[1+e^{0.2∙({opt}_{x}-10-{op}_{x})}\right]}\right\}∙\left\{\frac{1}{\left[1+e^{0.3∙(-{opt}_{x}-10+{op}_{x})}\right]}\right\}$$ (16-5)

and

$$\varepsilon_{x}=0.5+0.5∙\frac{\left\{[0.29K^{\frac{1}{2}}+0.6][K∙f(K)+0.469∙K^{\frac{1}{2}}+9.33∙{\left(\sum \theta \right)}^{-1}]\right\}}{\{\left(K+0.469∙K^{\frac{1}{2}}+0.966)[f(K)+0.933∙K^{-1}]\right\}}$$ (16-6)

where *SOL*_*x*_ is the total solar radiation, *SR*_min(*x*)_ represents the unvegetated land areas and is set to1.08, the value of *SR*_max(*x*)_ is related to the type of vegetation and has a range of 4.14–6.17, *opt*_*x*_ is defined as the mean temperature in the month when the NDVI reaches its annual maximum, *op*_*x*_ is the average annual temperature, *K* is the moisture coefficient, and *f*(*K*), *SR*_*x*_ is given as:

$${SR}_{x}=(1-{NDVI}_{x})/(1+{NDVI}_{x})$$ (16-7)

$$f(K)=K+0.906∙K^{\frac{1}{2}}+0.22$$ (16-8)

K=P/(0.1∙∑θ) (16-9)

Where *NDVI*_*x*_ is the normalized difference vegetation index, *P* is the annual precipitation, ∑*θ* is the active accumulated annual temperature (>0°C).

#### (17) Soil retention

The soil retention service mainly depends on the vegetation cover and root system [43]. This paper evaluates the benefits of soil retention service by a combination of the universal soil loss equation and outcome parameter method. The equations are as follows [30, 44]:

$$V_{x}=V_{x1}+V_{x2}+V_{x3}$$ (17-1)

$$V_{x1}=\sum SC∙Y_{k}∙T_{k}$$ (17-2)

$$V_{x2}=SC÷\vartheta ÷0.6∙(B/10000)$$ (17-3)

and

$$V_{x3}=SC÷\vartheta ÷24\%∙C$$ (17-4)

where *V*_*x*_ is the soil retention value provided by land-use type *x* per area annually, *V*_*x*1_ is a value of soil fertility maintenance, *V*_*x*2_ is a value of sediment deposition reduction, *V*_*x*3_ is the value of derelict land reduction, *SC* is the amount of soil conservation, *T*_*k*_ is the contents of nitrogen, phosphorus, potassium, and organic matter, *T*_*k*_ is the price of ammonium phosphate, potash fertilizer, and organic fertilizer, *k* stands for nitrogen, phosphorus, potassium, and organic matter, *ϑ* is the soil bulk density, *B* is the annual production value of land-use type *j* per unit area, *C* is the construction cost of a unit reservoir storage. *SC* is given as:

$$SC={SC}_{1}-{SC}_{2}$$ (17-5)

$${SC}_{1}=RE∙SE∙LS$$ (17-6)

$$A_{r}=RE∙SE∙LS∙VC∙PM$$ (17-7)

Where *SC*_1_ is the amount of potential soil erosion, *SC*_2_ is the amount of actual soil erosion, *RE* is the rainfall erosivity factor, *SE* is the soil erodibility factor, *LS* is a slope length and steepness factor, *VC* is a vegetation cover factor, and *PM* is a soil conservation measure factor. *RE*, *LS*, and *SE* are given as:

$$RE=\sum_{i=1}^{12}\left[1.735∙10^{1.5∙lg(P_{mean}^{2}/P)}\right]$$ (17-8)

$$LS={\left(L/20\right)}^{m}∙{\left(SG/10\right)}^{n}$$ (17-9)

$$SE=\left\{0.2+0.3∙e^{\left[0.0256∙SAN∙(1-\frac{SIL}{100})\right]}\right\}∙{\left[SIL/(CLA+SIL)\right]}^{0.3}∙[1-0.25∙\varphi /\varphi +e^{\left(3.75-2.59∙\varphi \right)}]∙\left\{1-0.7∙SNI/[SNI+e^{\left(22.9∙SNI-5.51\right)}]\right\}$$ (17-10)

SNI=1−(SAN/100) (17-11)

where, *P*_*mean*_ is the monthly precipitation, *P* is the average annual precipitation, *L* is the slope length, *SG* is the slope gradient, *m* and *n* are Chinese regional experience values, *SAN* is the content of sandy soil, *SIL* is the content of silt, *CLA* is the content of clay soil, *φ* is the content of organic matter. For regional empirical values setting, refer to Li [44].

#### (18) Disturbance prevention

A disturbance prevention service ameliorates ‘natural’ hazards and disruptive natural events caused ecological processes at the earth surface. It is computed as:

$$V_{x}=V_{mean(x)}∙f(v_{index(x)},\delta_{x})$$ (18-1)

where *V*_*x*_ is the value of disturbance prevention provided by land-use type *x* per area, *V*_mean(*x*)_, a global statistical value of TEEB, which is the average value of disturbance prevention provided by land-use type *x* per area, *v*_index(*x*)_ is the vulnerability index (0~1) of land-use type *x*, and *δ*_*x*_ is the resilience (0~1) of land-use type *x*.

#### (19) Waste treatment

Waste treatment service means that ecosystem store and recycle certain amounts of organic and inorganic human waste through dilution, assimilation and chemical re-composition. As its value cannot be estimated directly, an avoided cost is recruited to evaluate it according to the following equations [45, 46]:

$$V_{x}=V_{x1}+V_{x2}$$ (19-1)

$$V_{x1}=\sum \left(Y_{h}T_{h}\right)$$ (19-2)

$$V_{x2}=\sum \left(Y_{g}T_{g}\right)$$ (19-3)

Where *V*_*x*_ is the waste treatment value provided by land-use type *x* per area, *V*_*x*1_ is the atmospheric cleaning value, *V*_*x*2_ is the water cleaning value, *Y*_*h*_ is the amount of contaminant(i.e., sulfur dioxide, fluoride, nitrogen oxides, suspended dust) absorbed, *T*_*h*_ is the contaminant treatment expense, *h* stands for sulfur dioxide, fluoride, nitrogen oxides, and suspended dust, *Y*_*g*_ is the average amount of nitride and pnictide absorbed by water, *T*_*g*_ is the treatment expense of nitride and pnictide per unit of sewage, *g* stands for nitride and pnictide. For purification capacity parameter settings, refer to Zhang and Qian [45, 46].

## 4 General situation of the case area and data sources

Mentougou District is known as the western gate of Beijing and is located in the transition zone between the North China Plain and the Inner Mongolia Plateau. About 98.5% of its territory is mountainous. The topography of Mentougou District slopes from north-west to south-east. Mentougou has a rich diversity of geomorphy, consisting of middle and low-altitude mountains, river valley terraces, and floodplains, which create regional microclimates and spatial soil variations. With up to 70% of its territory covered with forest and grass, Mentougou District is both an important ecological shelter and water protection area for Beijing and is an ideal space for leisure and tourism. Mentougou District is also known as the western backyard garden of Beijing. It is a key region for the sustainable development of Beijing but it also faces serious conflicts between regional economic and ecological land users.

To calculate Mentougou’s land-use benefits, four types of data were used in this paper: land data, remote sensing data, socioeconomic data, and ecosystem monitoring data. Land use data, land price data, and *Regulation for gradation on agriculture land quality of China GBT28407-2012*. were collected from Beijing Municipal Commission Mentougou substation of Planning and Natural Resources (http://ghzrzy.beijing.gov.cn) [47, 48]. Soil data and ecosystem monitoring data were obtained from the National Science and Technology Infrastructure of China (http://www.cnern.org/index.action) [49, 50]. Meteorological data were derived from China Meteorological Data Service Center(http://data.cma.cn/en) [51]. Digital Elevation Model (DEM), solar radiation data, and other remote sensing data were mainly obtained from USGS Earth Resources Observatory and Science (EROS) Center (http://eros.usgs.gov/#) and China Meteorological Data Service Center (http://data.cma.cn/en) [52, 53]. Socio-economic data were retrieved from *Beijing Mentougou Statistical Yearbook* (2011), *Beijing Statistical Yearbook* (2011), *China Yearbook of Agricultural Price Survey* (2011), *Price Yearbook of China* (2011), *China Water Conservancy Yearbook* (2011), *China Forestry Statistical Yearbook* (2011), the Agricultural Information Network of China (http://www.agri.cn/), and *China Air Emissions Tariffs* [54–61]. Several types of statistical values of the ecosystem services were derived from the TEEB official database (https://www.cbd.int/incentives/teeb/) [62].

## 5 Results

The results of land-use benefits for each land-use type in Mentougou District are presented in Table 2. In land-use planning, many different demands for often-limited land must be weighed against each other. In this weighing process, the land-use benefits of each land-use type play an important role. Stakeholder appeal must be taken into account in the analysis and selection of land-use planning [63, 64]. Woodland, grassland, and water conservancy land can reap the highest ecological benefits. On the basis of *Mentougou Zoning Planning (Territorial Space Planning) (2017–2035)*, Mentougou is defined as an ecological conservation district in the western part of the capital Beijing. Therefore, to boost the production capacity of eco-products, ecological control zone is designated, the exploitation of forest and grassland is strictly restricted, an ecological control zone is designated, the exploitation of forest and grassland is strictly restricted, and ecological reconstruction of abandoned mining land and the harnessing of river channels are promoted. Scenic sites and special land can deliver an attractive balance among social, economic, and ecological land-use benefits and achieve a high level of comprehensive land-use benefits. As an ideal space for leisure and tourism for Beijing, tourism auxiliary facilities are improved and land in a suitable position would be developed into scenic sites to boost the quality of the tourism industry. In addition, transportation benefits are one kind of land-use benefit that is indispensable to prosperity and economic development. They can only be obtained from transportation land. In Mentougou District, the supply of transportation land is being increased to increase the transportation benefits according to the land-use planning *Mentougou Zoning Planning (Territorial Space Planning) (2017–2035)*.

**Table 2:** Results of land-use benefits evaluation in Mentougou District (Unit:yuan/hm^2^·a).

Land-use benefit type		Cultivated land	Garden land	Woodland	Grassland	Urban land	Mining land	Rural residential land	Scenic sites and special land	Transportation land	Water conservancy facilities land	Other land
Land-use economic benefits	Water	688.0	229.3	229.3	458.7	0	0	0	179.5	0	5351.4	458.7
	Food	12736.6	12736.6	1721.9	4743.9	0	0	0	0	0	69.4	22095.2
	Medicinal resources	0	272.5	0	0	0	0	0	0	0	0	0
	Raw materials	747.5	168.2	1083.9	1046.5	0	0	0	430.6	0	429.8	56.1
	Ornamental resources	0	0	922.9	0	0	0	0	0	0	922.9	922.9
	Energy minerals	0	0	0	0	0	1485455.7	0	0	0	0	0
	Products and services	0	0	0	0	1385836.4	0	0	0	0	0	0
	Total	14172.1	13406.6	3958.0	6249.1	1385836.4	1485455.7	0	610.1	0	6773.5	23532.8
Land-use social benefits	Habitation	0	0	0	0	177653.4	0	252095.2	0	0	0	0
	Transportation	0	0	0	0	0	0	0	0	145398.6	0	0
	Recreation and aesthetic information	10864.6	10864.6	13690.6	12569.0	296.0	0	81.0	18963.2	0	29877.7	24929.0
	Historic information	100.4	100.4	4408.1	7893.7	133.0	0	97.0	53107.3	0	2369.1	0
	Science, education, cultural and artistic information	5036.8	5036.8	27739.8	16983.0	421.0	0	110.0	43691.4	0	13642.9	17693.1
	Employment Security	103.5	103.5	103.5	103.5	484844.1	399691.6	0	67244.2	27050	103.5	53.5
	Basic living security	5500.0	4730.0	4048.0	4730.0	0	0	0	0	0	4048.0	4400.0
	Total	21605.3	20835.3	49989.4	42279.2	663347.5	399691.6	252383.2	183006.1	172448.6	50041.2	47075.6
Land-use ecological benefits	Gas regulation	12740.1	12740.1	13447.9	7431.7	0	0	0	3410.2	0	7618.3	2602.7
	Climate regulation	12068.7	12068.7	2769.8	34.5	0	0	0	1349.9	0	11948.9	2712.6
	Water regulation	4124.0	4475.2	11936.0	3659.8	0	0	0	4639.5	0	34343.6	1193.6
	Nutrient regulation	1960.2	1960.2	2068.1	1143.4	0	0	0	524.7	0	1172.2	400.5
	Pollination	1655.8	1655.8	17.2	0	0	0	0	0	0	0	64.7
	Species diversity	517.9	517.9	10134.8	5066.3	0	0	0	1674.3	0	6075.0	97.7
	Net primary productivity	4989.6	4989.6	5266.8	2910.6	0	0	0	1335.6	0	2983.7	1019.3
	Soil retention	27410.9	27410.8	121850.0	145059.4	19179.9	34483.4	39900.2	64483.2	35955.5	40663.8	13293.6
	Disturbance prevention	932.0	932.0	983.7	543.6	0	0	0	249.5	0	557.3	190.4
	Waste treatment	196	803.1	2732.7	86.2	0	0	0	506.1	0	106.2	13.2
	Total	66595.1	67553.5	171207.14	165935.6	19179.9	34483.4	39900.2	78172.9	35955.2	105468.9	21588.2
Land-use comprehensive benefits		102372.5	101795.4	225154.4	214463.8	2068363.8	1919630.7	292283.4	261789.1	208403.8	162283.6	92196.6

*Exchange Rate: 1$ = 6.755 yuan in 2010 [65]

In addition, land-use planning analysis and selection procedures are also bounded by natural conditions, location, culture, economy, and other internal and external factors. The plain, with high land-use social benefits, economic benefits, and comprehensive benefits, is entirely located in the east of Mentougou District, which point to the fact that it is the location of the local government as well as the regional business center. This phenomenon can be related to objective requirements of regional socioeconomic development [66]. There is a coupled or synergistic effect of economic and social land-use benefits. Often, economic sectors tend to have better employment security capability. Besides, income growth also influences human livelihoods, healthcare and education capacity, and associated facilities for scientific research, which generate good social benefits. Then, the improvement of regional social benefits undoubtedly improves production conditions and leads to a further agglomeration of factors of production, further promoting regional economic benefits. The middle and low mountain areas in the central and west regions of Mentougou District, which are mainly planted trees and grasses, are the main source of regional ecological benefits. The diverse landscape of middle and low mountain areas,95% of Mentougou District, has created regional microclimates that are suitable for the growth of trees and grass.

## 6 Discussion

### 6.1 Interrelationships of land-use benefits

Although each kind of land-use benefit was evaluated separately, they may be affected by multiple other land-use benefits. In our study, dependency relationship, superposition relationship, and repulsion relationship were found to be three main types of interrelationship among land-use benefits.

Dependency refers to the agglomeration, adsorption, synergy, and scale effects of land-use activities. In the natural world, an ecosystem’s habitat functions, production functions, and information functions depend on its regulation functions, which is an important theoretical foundation for the implementation of engineering measures. The delimitation of nature reserves and the implementation of reforestation projects in semi-arid areas, for example, are practical applications of dependency [15, 67, 68]. Modified dependency is an important way to increasing the diversity of land-use activities and plays a key role in regional socioeconomic development [69]. The key to modified dependency relationship is the spatial layout of land-use types, which is an important guideline to scientific socioeconomic measures; e.g., for urban planning, the layout of industrial and agricultural production, urban and rural planning. For example, building business and financial centers and accommodation and catering sites near urban settlements is conducive to agglomeration, adsorption, synergy, and scale effects.

Superposition relationship is similar to dependency relationship, but has own its traits, and highlights spatial sharing. The key to a superposition relationship is to eliminate the incompatible land-use benefits and embed other potentially compatible land-use benefits in the same land-use type. The main aspects are: (*i*) It should be recognized that a single land-use type can provide different kinds of land-use benefits. For example, wetlands provide valuable ecological benefits to human society [18]. These benefits consist of species diversity, climate regulation, water regulation (*ii*) It is a very important goal to alleviate land-use conflicts to transform enclosed spaces into open, active, and compatible spaces [70]. For example, a scenic spot’s space should be shared with the local residents.

A repulsion relationship is based on exclusive land occupation. exclusive land occupation. It pays greater attention to a single kind of land-use benefit. There are multiple objective requirements for repulsion relationships, ranging from defending land property rights and caring for human health and safety to safeguarding ethics and general welfare. Yet, repulsion relationship inadvertently and directly triggers the low-density land development and the spatial sprawl of artificial buildings. For example, the replacement of arable land and forest land by residential land directly displaces and renews all the kinds of land-use benefits, but low-density rural residential land has exacerbated regional land-use conflicts.

### 6.2 Advantages and limitations of the land-use benefit evaluation system

In practice, the land-use benefit evaluation systems that simply combine single indicators have various advantages, including low data quality requirements, low professional knowledge requirements for their users, acceptable operating costs, and the realization of policymakers’ goals [10, 14, 71]. Yet, these land-use benefit types also own disadvantages. Logic analysis, especially when all the land-use benefits of the same level are listed in detail, can become easily confused, and some kinds of land-use benefits cannot be easily measured by simple indicators. Although these land-use benefit evaluation systems can reflect the main goals of policymakers in practical applications, they may lead to unsustainable development, especially if policymakers neglected other reasonable land-use benefits, such as soil retention [17].

Our study was devoted to making a comprehensive system for evaluating the benefits of all land-use types. The study inherits some advantages of traditional research and the latest research results of the ecosystem services framework. It is necessary to emphasize one potential assumption, as well as the biases derived from the evaluation results. The potential hypothesis is that the land-use benefits of the same land-use types in the same zone are at the same. This potential hypothesis makes full use of statistical data in the calculation and simplifies the calculation. However, it ignores the spatial heterogeneity of land. Generally speaking, the goods and services produced by land in different geographical locations differ according to the differences in the soil characteristics and environment. In addition, biophysical models, equivalent factor methods, direct market valuation, and marketing value methods are proposed to meet the needs of land-use benefit evaluation [22, 41, 72]. Land-use economic benefits are often estimated by direct market valuations and equivalent factor methods, while land-use social and ecological benefits are most estimated by biophysical models, equivalent factor methods, and indirect market valuation. However, it is worth pointing out that there are still large uncertainties in the current research of land-use benefits due to the limitations in the theories, scientific knowledge, and technologies. Significant uncertainties in biophysical models persist in terms of data quality, parameter settings, and model applicability. Marketing value methods are considered more reliable, whereas indirect marketing value methods, such as contingent valuation and group valuation, are typically subjective. The use of equivalent factor methods is simple, but they have some drawbacks; e.g., spatial heterogeneity is ignored, high levels of professional knowledge are required by their operators, and they can be expensive to implement. Therefore, equivalent factor methods are not broadly adopted by policymakers [4].

## 7 Conclusion

In this paper, the land-use benefit classification system consisting of three primary types and 24 secondary types of benefits was reconstructed. Drawing on relevant research on evaluation of land functions, ecosystem services, and landscape functions, the evaluation function group of land-use benefits is systematically integrated. It is split into seven functional subsets of land-use economic benefits, seven functional subsets of land-use social benefits, and ten functional subsets of land-use ecological benefits.

M An example of the application of land-use benefits analysis is given based on a case study done in Mentougou District. The empirical result suggests that the evaluation result can precisely reflect the economic, social, and ecological benefits of each land-use type. Meanwhile, it also found that topography of Mentougou District determines the distribution of land-use benefits. Land-use economic benefits per area in the eastern plain are high, while the woodland and grasslands, which are dominated by mesic and low mountain landscapes, are the greatest contributors to regional ecological benefits. Synergetic effects between land-use economic and social benefits were found. The above results provide an aid for land resources managers in Mentougou District.
